# A Pilot Feasibility Study of a Group‐Based Remote‐Delivered Dyadic Exercise Intervention in Hispanic Men With Prostate Cancer and Their Caregivers

**DOI:** 10.1002/cam4.71709

**Published:** 2026-03-08

**Authors:** Meghan B. Skiba, Juan Contreras, Marjorie A. Nelson, Alejandro Recio‐Boiles, David O. Garcia, Floyd H. Chilton, Chris Segrin, Terry A. Badger, Kerri M. Winters‐Stone

**Affiliations:** ^1^ Nursing and Health Sciences Division College of Nursing, University of Arizona Tucson Arizona USA; ^2^ University of Arizona, Cancer Center Tucson Arizona USA; ^3^ Clinical Translational Sciences, College of Medicine University of Arizona Tucson Arizona USA; ^4^ Division of Hematology and Oncology, College of Medicine University of Arizona Tucson Arizona USA; ^5^ Department of Health Promotion Sciences Mel and Enid Zuckerman College of Public Health, University of Arizona Tucson Arizona USA; ^6^ Department of Nutritional Science and Wellness College of Agriculture, Life, and Environmental Science, University of Arizona Tucson Arizona USA; ^7^ Department of Communication College of Social and Behavioral Sciences, University of Arizona Tucson Arizona USA; ^8^ Department of Oncological Sciences, Knight Cancer Institute Oregon Health & Science University Portland Oregon USA

**Keywords:** exercising together, physical activity, physical function, psychosocial health, resistance training

## Abstract

**Purpose:**

The purpose of this study was to determine the feasibility and acceptability of a Hispanic adapted culturally relevant *Exercising Together^©^* intervention (HACER) through a single‐arm pilot in post‐treatment Hispanic men with prostate cancer and their caregivers.

**Methods:**

Dyads participated together in a live, remote group‐based 12‐week exercise intervention with resistance training three times a week. Primary outcomes were intervention feasibility and acceptability. Secondary outcomes were measures of physical and psychosocial health. Assessments were completed at baseline and post‐intervention. Mean difference and effect sizes were calculated to determine intervention effects.

**Results:**

Accrual of eligible dyads was 75%. Fourteen eligible dyads were allocated to intervention. Attendance averaged 62%. Retention was 79% with 95% assessment completion. Participants rated HACER as acceptable and 86% would recommend the program to other dyads. Self‐reported physical activity level and objective physical function improved for survivors and caregivers, with clinically significant improvement in caregiver depressive symptoms.

**Conclusions:**

HACER was feasible and acceptable with modest improvements in physical and psychosocial health, especially for Hispanic survivors. HACER shows promise as a scalable intervention to improve health outcomes for Hispanic cancer survivors and caregivers when they train together; a larger, fully powered efficacy trial is warranted.

**Abbreviations**
ACSM: American College of Sports Medicine
ADT: Androgen deprivation therapy
ARMSA‐II: Acculturation Rating Scale for Mexican Americans
BMI: Body mass index
CRA: Caregiver Reaction Assessment
FACT‐G: Functional Assessment of Cancer Therapy—Prostate
FACT‐P: Functional Assessment of Cancer Therapy—General Caregiver
HR‐QOL: Health‐Related Quality of Life
ICIQ‐IU SF: International Consultation on Incontinence Questionnaire‐Urinary Incontinence Short Form
MCID: Minimal clinically important difference
PROMIS: Patient Reported Outcomes Measurement Information System
RAPA: Rapid Assessment of Physical Activity
RCT: Randomized controlled trial
SPPB: Short Physical Performance Battery

## 1 Introduction

Prostate cancer is the most common cancer among Hispanic men, who are disproportionately diagnosed with advanced prostate cancer [1, 2]. Compared to non‐Hispanic whites (NHWs), Hispanics experience lower prostate cancer‐specific mortality but greater comorbidity risk and lower quality of life [2, 3]. Improvements in cancer screening, diagnosis, treatment, and supportive care have led to increased survival, increasing the number of survivors living with long‐term side effects [4]. Prostate cancer treatment can result in deleterious health consequences (e.g., reduced physical function) that contribute to morbidity and mortality burden [5, 6, 7, 8]. Informal cancer caregivers, individuals who provide unpaid support and care of survivors [9], also experience poor health and unmet needs [10, 11, 12]. For both the survivor and caregiver, physical activity can mitigate cancer treatment side effects such as fatigue and weakness and improve overall quality of life [13, 14], however, many survivors and caregivers are inactive [15]. The health of the cancer survivor and the caregiver are intertwined [16]; thus, when caregivers experience poor health, survivors also experience negative health outcomes [17]. Recent evidence suggests interventions including the caregiver can better improve adherence to physical activity guidelines and improve cancer outcomes [18], while also benefiting the caregiver [19]. Few dyadic physical activity interventions are available for Hispanic cancer survivor‐caregiver dyads.

Current guidelines from the American College of Sports Medicine (ACSM) recommend prostate cancer survivors engage in regular physical activity including resistance training at least twice a week after cancer treatment; a level of activity that favorably improves fatigue, physical function, and quality of life [20]. For most men, engagement in physical activity decreases during prostate cancer treatment and does not return to pre‐diagnostic levels [21]. Sociocultural influences, family dynamics, and cultural values are influential to physical activity engagement among Hispanic men [22], necessitating culturally relevant interventions to promote physical activity in this population. Further, culturally relevant physical activity interventions including both the survivor and their caregiver may improve health and reduce the morbidity burden experienced in Hispanic populations [23].

*Exercising Together* ^©^ is an evidenced‐based dyadic health management strategy of partnered progressive resistance training. Originally designed for survivors and their spouses to promote teamwork and collaboration and buffer the effects of cancer on the intimate relationship [24, 25], it has demonstrated positive impacts on the physical, mental, and relational health of primarily NHW prostate cancer survivors and their spouses [26, 27]. We adapted *Exercising Together* ^©^ through stakeholder engagement in efforts to expand the reach and scalability of the intervention and address the existing gap in available dyadic exercise interventions for Hispanic cancer survivors and their caregivers [28]. However, the feasibility and acceptability of this intervention (Hispanic‐adapted and culturally relevant *Exercising Together^©^* [HACER]) remains undefined in this population. Consequently, there is a need for pilot testing prior to conducting an efficacy trial.

The present study examined: (1) the feasibility and acceptability of HACER for Hispanic men with prostate cancer and their caregivers; and (2) the effect of HACER on the physical and psychosocial health of the dyads.

## 2 Methods

### 2.1 Study Design and Setting

We conducted a single‐arm pilot study of an adapted and culturally relevant *Exercising Together^©^* for Hispanic cancer survivors and caregivers (HACER), in a sample of 14 Hispanic men with prostate cancer and their caregivers to determine feasibility and acceptability. This live, remote study was centrally conducted in Tucson, Arizona and received Institutional Review Board approval (STUDY00003138) from the University of Arizona. All participants provided informed consent prior to beginning study procedures. The trial was registered on clinicaltrials.gov (NCT06018311).

### 2.2 Study Sample

Our target sample size was 15 survivor‐caregiver dyads (*N* = 30 total participants). A formal power analysis for this study was not completed, as the aim was to determine effect size estimates for planning a subsequent efficacy trial [29]. Hispanic men with prostate cancer and their caregivers were enrolled from April 2024 to September 2024 on a rolling basis. Participants were recruited locally from direct provider referrals, community outreach, and flyers in healthcare facilities. An online recruitment vendor (BuildClinical, OpenClinica, New York, NY, USA) was also utilized to expand geographic reach for increased generalizability. Study staff screened individuals via phone to determine interest and eligibility and to obtain informed consent.

To be eligible, both survivors and caregivers had to: (1) be ≥ 18 years of age at time of participation; (2) have the ability to read, speak, and understand English or Spanish; (3) provide informed consent; (4) have access to a mobile device with camera and internet; (5) be willing to attend group exercise sessions and complete study assessments; (6) be able to ambulate with or without assistive devices; (7) have no contraindication to participation in moderate intensity exercise; and (8) be able to attend > 75% of scheduled exercise class times. Survivors and caregivers were required to participate in the group exercise classes at the same time in the same location. Physician clearance to participate was required if indicated by the pre‐participation screening criteria from the ACSM [30]. Additional eligibility criteria for survivors included: (1) self‐identified as Hispanic; (2) history of prostate cancer; (3) completed cancer treatment with curative intent (i.e., chemotherapy, surgery, radiation) ≥ 6 weeks prior to enrollment; and (4) identified a caregiver of any relationship who can participate with them. There were no treatment restrictions and survivors were eligible if they were on hormonal (i.e., androgen deprivation therapy [ADT]) or maintenance therapy. Caregivers did not have to self‐identify as Hispanic to be eligible.

### 2.3 Intervention

HACER was adapted from the original *Exercising Together^©^* progressive dyadic resistance training program. Our adaptation included six key adaptions [28]: (1) exercise dose of thrice‐weekly over 12‐weeks (instead of twice weekly over 6‐months); (2) both synchronous and asynchronous exercise sessions led by bilingual and bicultural Hispanic interventionists; (3) available in English or Spanish; (4) entirely remote administration and assessment; (5) inclusion of the survivor identified caregiver; and (6) minimization of spousal‐caregiver specific elements in exercises (i.e., intimacy building).

The dyadic resistance training curriculum was grounded in the principles of functional exercise based on recommendations for muscle strength, safe for cancer survivors [31], and was designed to establish teamwork and collaboration [24, 25]. Each participant received an exercise prescription that included achievable goals with progression tailored based on individual capacity and tolerance [20, 31]. Live exercise sessions were delivered twice weekly synchronously via Zoom (Zoom Video Communications Inc., San Jose, CA, USA), by certified bilingual exercise instructors trained on intervention protocols with class sizes ranging from 3 to 5 dyads. The third session each week was asynchronous. Participants were sent a recording of the second session each week through a private YouTube link. Each exercise session included: (1) teamwork building exercises for the dyad; (2) dynamic warm‐up exercises; (3) resistance exercises; and (4) cool‐down stretches. The program had a specific focus on muscle groups and movements used in activities of daily living such as squats, chair stands, lunges, rows, push‐ups, bridges, and planks. Teamwork was emphasized through tandem versions of exercises which required collaboration and communication (e.g., squats performed face‐to‐face). During the resistance exercises, each member of the dyad rotated between a ‘coach’ and an ‘trainee’ role, which provides built in rest between sets as only one person was exercising at a time (unless a tandem exercise) and further integrated communication and collaboration into routine exercises.

### 2.4 Study Procedures

Prior to beginning the intervention, participants completed an exercise intake questionnaire that was provided as a summary to exercise instructors to inform exercise prescriptions and progression. Assessments were completed at baseline prior to beginning intervention (T1) and at post‐intervention (T2). Participants were mailed a set of three resistance bands (TheraBand CLX in Black, Siver, and Gold) and adjustable dumbbells (PowerBlock Sport 24) to their home address after completion of T1 assessments and were able to keep the equipment at intervention completion to support retention. In an effort to improve adherence, participants received a class time reminder 24‐h before each session via email or text. All assessments were culturally competent, previously validated, and available in English or Spanish. For all self‐reported questionnaires, participants were asked to consider a recall period of the previous 7 days. After completion of assessments at each time point, participants received $10 USD compensation for their time.

### 2.5 Primary Outcomes

#### 2.5.1 Feasibility and Acceptability

Feasibility was measured by accrual (# of dyads enrolled/# dyads eligible), retention (# dyads completing intervention/# dyads enrolled), adherence (# exercise sessions attended/# exercise sessions prescribed), and assessment completion (# completed/# planned). Attendance to exercise sessions was documented by study staff during synchronous group sessions. For asynchronous sessions, participants reported completion as a dyad via individual online survey which was confirmed by YouTube link engagement. Feasibility benchmarks were set as ≥ 60% accrual, ≥ 75% retention, ≥ 50% adherence, and ≥ 80% assessment completion as used in previous exercise trials [24, 25, 26, 27]. Acceptability was assessed by evaluation survey specific to the intervention which included measures of participation satisfaction (10‐point Likert scale, 1 = poor; 10 = exceptional) and perceptions of intervention accessibility, effectiveness, and usability (4‐point Likert scale, 1 = strongly disagree; 4 = strongly agree). Participants additionally completed a validated 12‐item measure of intervention feasibility, acceptability, and appropriateness [32]. Safety of the intervention was measured by adverse event reporting. Participants self‐reported any adverse events once monthly through REDCap survey. An adverse event occurring in class was documented by study staff. After completion of all intervention activities and assessments, participants were invited to complete a semi‐structured exit interview via phone to provide additional feedback on participation.

### 2.6 Secondary Outcomes

#### 2.6.1 Physical Health

*Physical Activity* was measured using the Rapid Assessment of Physical Activity (RAPA), a validated, reliable, and culturally relevant measure of activity for older adults [33, 34]. The RAPA classifies aerobic physical activity into 5 categories (sedentary, underactive, underactive regular‐light, underactive regular, and active), with categories less than active considered suboptimal physical activity. The RAPA also classifies strength and flexibility physical activity into 4 categories (none, strength only, flexibility only, and both). Self‐efficacy for physical activity was measured using a 5‐item scale, with a greater score indicating greater self‐efficacy [35]. Dyads were asked about shared physical activities completed together using a 2‐item measure that rates engagement together in leisure activities and exercise activities on a 5‐point Likert scale (0 = none; 5 = a great deal).

*Physical Function* was assessed objectively using the Short Physical Performance Battery (SPPB), a gold‐standard and reliable measure consisting of three timed tests: five repeated chair stands, standing balance, and gait speed [36]. Each of the three tests was independently scored 0 (unable) to 4 (completes without difficulty), then summary scored. The possible range of scores is 0–12, with a higher score indicating higher physical functioning. An improvement in SPPB score of > 1 is considered the minimal clinically important difference (MCID) for older adults [37]. The SPPB was assessed remotely using previously established procedures with documented validity and reliability for cancer survivors and caregivers [38, 39]. After completing self‐report questionnaires, dyads were contacted via phone and asked to participate in an optional remote physical function assessment. If the dyad agreed, an assessment kit was mailed to their residential address with return postage paid. To standardize assessment across locations, the assessment kit included a tablet computer with videoconferencing software installed, a tripod stand, a 4‐m piece of rope, orange cones, and an instruction packet. The packet included a link to an online demonstration video that participants were asked to review before the assessment. Prior to beginning the remote assessment, a safety appraisal was completed with study staff. Using videoconferencing (Zoom), the SPPB was conducted by trained study staff with each test completed twice and the average of the two test scores used for analysis.

*Urinary Incontinence* symptom frequency, severity, and impact were measured, for prostate cancer survivors only, using the 4‐item International Consultation on Incontinence Questionnaire‐ Urinary Incontinence Short Form (ICIQ‐IU SF) that has demonstrated good validity and reliability [40]. Survivors were also asked an additional question of “How many times do you change a pad, diaper, or other sanitary protection during a typical 24‐hour period?”

#### 2.6.2 Psychosocial Health

*Anxiety and Depression Symptoms* were measured using the Patient Reported Outcomes Measurement Information System (PROMIS) Anxiety and Depression 8‐item short forms [41]. These measures have demonstrated high reliability Hispanic cancer survivors and caregivers [42]. For each item, participants reported their experience on a 5‐point Likert scale (1 = never; 5 = always). PROMIS measures for anxiety and depression were each scored using T‐metrics; a higher T‐score indicates greater depression or anxiety symptoms. Population mean T‐score is 50 with a standard deviation of 10. The MCID for PROMIS measures is 3–4 points [43, 44].

*Health‐Related Quality of Life (HR‐QOL)* was measured using the 39‐item Functional Assessment of Cancer Therapy—Prostate (FACT‐P) for survivors [45] the 27‐item general FACT for caregivers (FACT‐G) [46]. Four domains of HR‐QOL are assessed, including physical well‐being, social/family well‐being, emotional well‐being, functional well‐being. The FACT‐P has a prostate cancer specific subscale. Items are on a 5‐point Likert scale (0 = not at all; 4 = very much) with a score range of 0–156 for FACT‐P and 0–108 for FACT‐G. Higher scores indicate better HR‐QOL. The MCID for FACT‐P and FACT‐G is 6–10 points and 3–7 points, respectively [47, 48].

*Relationship Health* was measured for both the survivor and caregiver using the Relationship Quality Index (RQI), a 6‐item assessment of communication and satisfaction which has been used in dyads composed of various relationships [49]. Items were asked on a 7‐point Likert scale (1 = very strongly disagree; 7 = very strongly agree) and summary scored with higher scores indicating higher quality relationships. Strain was measured for caregivers using the 24‐item Caregiver Reaction Assessment (CRA) [50]. The CRA has good reliability and validity to measure the impact of cancer caregiving on five domains (schedule, finances, family, health, and self‐esteem) on a 5‐point Likert scale (1 = strongly disagree and 5 = strongly agree). Responses were summary scored with higher scores indicating greater strain.

### 2.7 Sociodemographic and Clinical Characteristics

Sociodemographic variables, including age, sex, ethnicity, origin, race, education, occupation, health insurance status, income, relationship status, and household size were collected by self‐report at T1. For survivors only, cancer (i.e., stage) and treatment information (i.e., radiation, ADT) were obtained by self‐report. Comorbid conditions were reported using the Charlson Comorbidity Index, a weighted index developed originally to predict mortality [51, 52], modified to exclude cancer. Participants provided self‐reported current medications, smoking history, and alcohol use as well as height and weight which was used to calculate body mass index (BMI).

### 2.8 Analytical Procedures

Descriptive statistics of feasibility and acceptability primary outcomes were completed and reported as means or frequencies. Effect sizes were calculated from T1 to T2; Cohen's *d* for effect sizes were used (0.2 = small; 0.5 = medium; 0.8 = large) [53]. Paired *t*‐tests were conducted to compare change of secondary outcomes between T1 and T2 and compared to MCID metrics. Due to the pilot nature of this study and small sample size, analysis did not focus on statistical significance but on effect size and MCID. Statistical analyses were completed using STATA 18.0 (College Station, TX, USA). Interviews were transcribed verbatim in the native language and analyzed by two coders using Excel Version 16.99.2. (Microsoft Corporation, Redmond, WA) following a rapid analysis approach [54].

## 3 Results

### 3.1 Participant Characteristics

Sociodemographic and clinical characteristics for enrolled survivors (*n* = 14) and caregivers (*n* = 14) are detailed in Table 1. Nine dyads were recruited locally from Southern Arizona; 5 dyads were recruited via online strategies and resided in Georgia, Illinois, New Jersey, New Mexico, or Texas. On average, participants were on average aged 62.4 ± 12.4 years at the time of participation. All dyads were co‐inhabiting. The majority were composed of spouse/partners (93%) with one dyad reporting friendship as the relationship type. All survivors identified as Hispanic males while most caregivers identified as female (93%) and Hispanic (64%). Among Hispanic identifying survivors and caregivers, Mexican origin was the most prominent (69.6%). Comorbidity burden and BMI were not significantly different between survivors and caregivers.

**TABLE 1:** Baseline sociodemographics of dyads allocated to intervention.

Mean (SD) or *n* (%)	Survivor (*n* = 14)	Caregiver (*n* = 14)	Total (*N* = 28)
Age (years)	65.3 (11.8)	59.5 (12.7)	62.4 (12.4)
Relationship length (years)^a^	26.6 (15.0)	26.0 (15.1)	26.3 (14.8)
Prostate cancer stage B			
I	1 (7.1%)	—	—
II	4 (28.6%)	—	—
III	2 (14.3%)	—	—
IV	1 (7.1%)	—	—
Unknown	6 (42.9%)	—	—
Cancer treatment^b^			
Surgery	10 (71.4%)	—	—
Radiation	7 (50.0%)	—	—
Chemotherapy	2 (14.3%)	—	—
Other	1 (7.1%)	—	—
Receiving ADT^b^	4 (28.6%)	—	—
Sex^d^			
Male	14 (100.0%)	1 (7.1%)	15 (53.6%)
Female	0 (0.0%)	13 (92.9%)	13 (46.4%)
Preferred language			
English	7 (50.0%)	8 (57.1%)	15 (53.6%)
Spanish	7 (50.0%)	6 (42.9%)	13 (46.4%)
Ethnicity identity^d^			
Hispanic	14 (100.0%)	9 (64.3%)	23 (82.1%)
Non‐hispanic	0 (0.0%)	5 (35.7%)	5 (17.9%)
Education			
≤ High school diploma/GED	6 (42.9%)	4 (28.6%)	10 (85.7%)
Associate degree or equivalent	2 (14.3%)	3 (21.4%)	4 (14.3%)
Bachelors degree	1 (7.1%)	3 (21.4%)	4 (14.3%)
Postgraduate/advanced degree	3 (21.4%)	4 (28.6%)	7 (50.0%)
Decline to answer	2 (14.3%)	0 (0.0%)	2 (7.1%)
Current employment status			
Retired	7 (50.0%)	8 (57.1%)	15 (53.6%)
Full time	5 (35.7%)	4 (28.6%)	9 (32.1%)
Part time	1 (7.1%)	1 (7.1%)	2 (7.1%)
Unemployed	1 (7.1%)	1 (7.1%)	2 (7.1%)
Health insurance			
Uninsured	0 (0.0%)	2 (14.3%)	2 (7.1%)
Medicare/Medicaid	7 (50.0%)	5 (37.5%)	12 (42.9%)
Private insurance	4 (28.6%)	4 (28.6%)	8 (28.6%)
Other	3 (21.4%)	2 (14.3%)	5 (17.9%)
Decline to answer	0 (0.0%)	1 (7.1%)	1 (3.6%)
Annual household income			
Less than $25,000	3 (21.4%)	4 (28.6%)	7 (25.0%)
$25,000 to $49,999	4 (28.6%)	1 (7.1%)	5 (17.9%)
$50,000 to $99,999	3 (21.4%)	3 (21.4%)	6 (21.4%)
≥ $100,000 to $149,999	4 (28.6%)	4 (28.6%)	8 (28.6%)
Decline to answer	0 (0.0%)	2 (14.3%)	2 (7.1%)
Current relationship status			
Married/partnered	12 (85.7%)	11 (78.6%)	23 (82.1%)
Other	2 (14.3%)	3 (21.4%)	5 (17.9%)
Body mass index, kg/m^2^	26.6 (3.5)	28.8 (5.5)	27.7 (4.7)
Charlson comorbidity index^c^	0.9 (0.8)	0.9 (1.2)	0.9 (1.0)

^a^ Asked for partnered dyads only.

^b^ Questions asked for survivor only.

^c^ Excluding cancer.

^d^ *p* < 0.05, all other *p* > 0.5.

### 3.2 Primary Outcomes

#### 3.2.1 Feasibility and Acceptability

**FIGURE 1:**
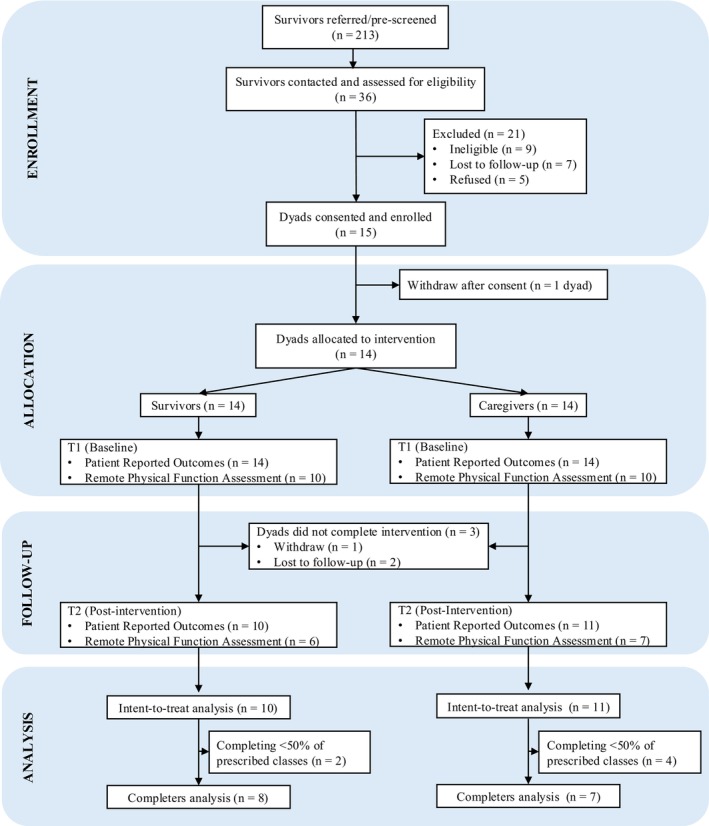
Consolidated Standard of Reporting Trials (CONSORT) diagram of participant flow through study procedures.

Participant flow through the study is depicted in Figure 1. The HACER intervention was delivered between April 2024 and December 2024 with three separate cohorts of dyads. All feasibility metrics were met. A total of 15 dyads consented of 20 eligible dyads, an accrual rate of 75%. One dyad withdrew after consent; therefore, 14 dyads were allocated to the intervention. One dyad withdrew in Week 1 and two dyads were lost to follow‐up, resulting in a 79% retention rate. Average dyad attendance to scheduled sessions was 62% overall. Attendance was lower for synchronous (59%) compared to asynchronous (69%) sessions. Of the 11 dyads completing intervention, 10 survivors and 11 caregivers completed post self‐report assessments: a 95% average assessment completion rate. No study‐related adverse events were reported.

Overall participation in HACER was satisfactory for participants, with scores slightly higher for caregivers than for survivors (8.1 ± 2.0 and 7.6 ± 2.7, respectively); 86% of participants would recommend the program to other cancer survivors and caregivers. Most participants (76%) preferred the virtual delivery of exercise classes. Overall perceptions of intervention accessibility, effectiveness, and usability were positive (Table 2), and the intervention was considered feasible (mean score: 4.3 ± 1.2), acceptable (mean score: 4.5 ± 0.6), and appropriate (mean score: 4.6 ± 0.6) by participants. Text responses on open‐ended questions and exemplar quotes from the interviews (*n* = 13) are in Table 3. Key themes included perceptions of positive support and flexibility, togetherness, and improvement in physical and emotional health.

**TABLE 2:** Perceptions of Intervention Accessibility, Effectiveness, and Usability.

Perception	Survivor (*n* = 10) mean (SD)	Caregiver (*n* = 11) mean (SD)
Accessibility		
“I felt safe in class”	3.8 (0.4)	3.8 (0.4)
“Class times fit my schedule”	3.4 (0.8)	3.3 (0.6)
“Free classes were a large incentive for me”	3.6 (0.5)	3.6 (0.7)
“Getting connected to virtual classes was easy”	3.6 (0.5)	3.4 (1.0)
“There was adequate space in my home to perform the exercises”	3.3 (0.7)	3.5 (0.7)
“Instructors were professional and credible”	3.9 (0.3)	3.9 (0.3)
“Instructors adapted/modified exercises to my needs”	3.8 (0.4)	3.8 (0.4)
Effectiveness		
“Classes helped motivate me to exercise”	3.8 (0.4)	3.7 (0.4)
“I felt the class exercises improved my health and my fitness”	3.6 (0.5)	3.7 (0.5)
“Instructors were encouraging and supportive”	3.9 (0.3)	3.9 (0.3)
“Instructors were knowledgeable about cancer”	3.6 (0.5)	3.7 (0.5)
“Instructors were a good role model for me”	3.7 (0.5)	3.9 (0.3)
Usability		
“I would use this frequently”	3.1 (0.7)	3.0 (0.4)
“The virtual classes were easy to use”	3.0 (0.5)	3.0 (0.6)
“I felt very confident with the virtual classes”	2.7 (0.6)	3.1 (0.5)
“The class environment was social and entertaining”	3.6 (0.5)	3.5 (0.5)
“I enjoyed exercising with my spouse/partner”	3.7 (0.5)	3.8 (0.4)
“I enjoyed the type of exercises done in class”	3.7 (0.5)	3.8 (0.4)

**TABLE 3:** Qualitative feedback of intervention participation from survivors and caregivers (*n* = 13).

Theme	Survivor comment	Caregiver comment
Positive Support and Flexibility	The team created and maintained a friendly atmosphere, one of good humor and understanding.	We enjoyed the classes but were not able to do some at the time [scheduled].
	I am very thankful for the support.	It was motivating.
	I can't go to the gym, but I like the class[es]; we work on the whole body.	The comfort of doing the exercises at home and doing them as a couple.^a^
	It helps us a lot, especially those of us who have this illness; it motivates us.^a^	A very pleasant environment and good exercises.^a^
	For an exercise program I thought it was easy to connect. Something to keep going. I had [time] blocked off for those days.	
	It's nice to be able to do it in [our] home.	
Togetherness	I thought it was very helpful for me and [partner] because we do enjoy doing things together.	It is a good program that allows a very doable set of activities which promote endurance and strength in both survivor and partner.
	We enjoyed doing it together. The exercises themselves were something we don't normally do.	It was nice to learn how to do exercises together that help improve strength and flexibility.
	We had a good time together.	The classes allow quality time with partners who need closeness and touch in their lives. Medical procedures sometimes take long to recover from and the closeness sometimes is put on hold. It was a positive experience for both my husband and I.
		Having cared for [survivor] and continuing to help care with him and for him, it just really stems to the support that we can give each other.
Improvement in Physical and Emotional Health	I am more flexible now because of classes.	Exercising was just a joy.
	It helped have better range of motion and strength. It also helped because it taught me about enjoying life despite a cancer diagnosis. Also, that we must do whatever we can to be healthy.	I am very happy and motivated to continue with the routine of exercising together because was my first time doing it. I am able to move more than before. The exercises are helping me with my joints and body aches. I can take the sweat now because before I hated it. The sweat used to bring bad memories from my childhood and now it's over it. I lost weight and inches in these 3 months we did the exercises which it's great for me.
	I really like and enjoy exercising because it helps me relax.	
	I feel more healthy and with more energy.	
	My mobility flexibility is much better now than it was.	

^a^ Spanish feedback was translated to English for publication.

### 3.3 Secondary Outcomes

#### 3.3.1 Physical Health

Physical health outcomes improved for dyads overall (Table 4). There was a significant and large effect on self‐reported strength physical activity, with the observed effect larger for caregivers than survivors (*p* = 0.04; *d* = −0.92). There were divergent effects of the intervention on physical activity self‐efficacy with a small decrease for survivors, but a negligible increase for caregivers. There was also a significant increase in reported shared exercise activities (*p* = 0.03), with 92% of participants increasing at least one level (i.e., “a little” to “some”). Ten dyads completed the SPPB at T1 and seven completed the SPPB at T2. There was a non‐significant medium effect of the intervention on SPPB scores, with the effect larger for survivors than for caregivers (*p* = 0.30; *d* = −0.56). Average survivor SPPB improvement was 0.7 points (95% CI: −2.1, 0.7), nearly reaching clinically meaningful improvements. There was not an observed effect on change in urinary incontinence for survivors.

**TABLE 4:** Survivor and caregiver physical and psychosocial health outcomes.

Mean (95% CI)	Survivor (*n* = 11)				Caregiver (*n* = 11)				Total (*N* = 22)			
	T1	T2^e^	*p*	Cohen's *d*	T1	T2	*p*	Cohen's *d*	T1	T2^e^	*p*	Cohen's *d*
Physical health												
Aerobic physical activity	4.5 (4.0, 4.9)	4.5 (3.8, 5.2)	0.9	−0.05	4.2 (3.5, 4.8)	4.3 (3.8, 4.7)	0.80	−0.11	4.3 (3.9, 4.7)	4.4 (4.0, 4.7)	0.80	−0.08
Strength physical activity	1.5 (0.5, 2.4)	2.1 (1.2, 3.0)	0.29	−0.46	1.1 (0.2, 2.0)	2.3 (1.5, 3.1)	0.04	−0.92	1.3 (0.7, 1.9)	2.2 (1.6, 2.7)	0.03	−0.70
Shared exercise	2.1 (1.1, 3.1)	3.1 (2.4, 3.8)	0.08	−0.55	2.2 (1.6, 2.8)	2.7 (2.0, 3.5)	0.21	−0.53	2.1 (1.6, 2.7)	2.9 (2.4, 3.4)	0.03	−0.69
Physical activity self‐efficacy	16.7 (13.9, 19.5)	15.1 (13.4, 18.4)	0.51	0.29	16.3 (13.2, 19.4)	16.5 (12.4, 20.5)	0.93	−0.03	16.5 (14.6, 18.4)	15.8 (13.0, 17.8)	0.66	0.13
Physical function (SPPB total score)^a^	7.3 (6.3, 8.3)	8.0 (7.1, 8.9)	0.30	−0.56	6.5 (5.0, 8.0)	7.0 (5.4, 8.6)	0.61	−0.26	6.9 (6.1, 7.7)	7.5 (6.6, 8.3)	0.35	−0.34
Urinary incontinence^b^	10.1 (6.6, 13.6)	9.8 (5.2, 14.4)	0.91	0.05	—	—	—	—	—	—	—	—
Psychosocial health												
Anxiety	48.8 (41.2, 56.3)	49.0 (40.0, 58.0)	0.95	−0.02	50.4 (43.1, 57.8)	48.9 (39.4, 58.5)	0.78	0.12	49.6 (44.8, 54.4)	49.0 (43.0, 55.0)	0.87	0.05
Depression	49.3 (42.4, 56.2)	49.7 (45.3, 53.6)	0.92	−0.04	49.6 (44.0, 55.2)	46.2 (39.6, 52.8)	0.38	0.40	49.5 (45.4, 53.5)	47.9 (43.9, 52.0)	0.59	0.17
HR‐QOL^c^	114.6 (101.3, 128.0)	113.4 (95.4, 131.4)	0.90	0.05	82.4 (74.2, 90.7)	79.6 (59.1, 100.0)	0.77	0.12	—	—	—	—
Relationship quality	27.2 (23.8, 30.6)	22.6 (15.5, 29.7)	0.21	0.55	23.1 (26.8, 29.4)	25.5 (20.4, 30.4)	0.52	−0.28	25.1 (21.7, 28.5)	24.0 (20.0, 28.1)	0.67	0.13
Strain^d^	—	—	—	—	12.3 (10.8, 13.7)	12.6 (10.8, 14.9)	0.59	−0.24	—	—	—	—

Abbreviations: T1‐ Baseline; T2‐ Post intervention; SPPB‐ short physical performance battery; HR‐QOL‐ Health‐related quality of life.

^a^ *n* = 7 dyads completed post SPPB; 3 unavailable to complete due to holiday schedules.

^b^ Questions only asked for survivors.

^c^ Survivor completed FACT‐P (score range 0–156) while caregiver completed FACT‐G (score range 0–108).

^d^ Questions only asked for caregivers; *n* = 1 caregiver declined response to questions in T2.

^e^ *n* = 1 Survivor declined post questionnaires.

#### 3.3.2 Psychosocial Health

The intervention had mixed effects on psychosocial health for survivors and caregivers (Table 4). Anxiety and depressive symptoms decreased overall, though not significantly across the intervention. Caregivers demonstrated greater improvement in these symptoms than survivors. There was a non‐significant small to medium effect on depressive symptoms among cancer caregivers that reached clinical significance (Δ = 3.4; 95% CI: −4.6, 11.4; *p* = 0.38; *d* = 0.40). There was no significant effect on HR‐QOL. Perception of relationship quality improved for caregivers but decreased for survivors. There was no change in caregiver strain.

## 4 Discussion

This study assessed the feasibility and acceptability of the Hispanic adapted and culturally relevant *Exercising Together^©^* (HACER) intervention for Hispanic men with prostate cancer and their caregivers. Our primary outcome was feasibility and acceptability. Specifically, we sought to determine recruitment and retention rates, adherence to the exercise classes, data collection completion rates, and participant satisfaction in preparation for a larger‐scale efficacy trial. Our secondary outcomes were to determine effect sizes for changes in physical and psychosocial health outcomes that are important to Hispanic cancer survivors and caregivers.

HACER was feasible and acceptable in a sample of 14 Hispanic prostate cancer survivors and their caregivers (*N* = 28 participants). Most dyads were composed of spouses/partners despite our broad definition for caregiver. This may be related to Hispanic cultural values wherein caregivers are often female family members [10]. All benchmarks for feasibility were exceeded for accrual, adherence, retention, and assessment completion. This may be attributed in part to the availability of the intervention in both English and Spanish and ethnically and gender‐matched interventionists. No study‐related adverse events occurred and the intervention was acceptable for both survivors and caregivers. During the 12‐week intervention, objective physical function improved, with greater effects for survivors, while caregivers experienced a greater effect on depressive symptoms.

We met our target enrollment, navigating recruitment challenges engaging an underrepresented population of cancer survivors. Refusal of participation was due to lack of interest in exercise, schedule conflicts, or required time commitment. Clinical referral of participants was successful but required extensive follow‐up from study staff, with many lost to follow‐up. Our retention rate (79%) may have been influenced by recruitment channel. Three of the four dyads that did not complete the intervention were recruited through online strategies, suggesting that the intervention may not have resonated in the same way or was not advertised appropriately. Conversely, retention from clinical referrals may have been higher due to reinforcement from healthcare providers [22, 55]. Our strategies for recruitment and retention were in alignment with principles previously identified to improve rates among underrepresented cancer survivors and caregivers in clinical research [56].

Participants attended 62% of all sessions, with 82% of survivors and 64% of caregivers completing > 50% of prescribed sessions, respectively. Adherence and attendance rates were most influenced by scheduling conflicts. Early in the intervention, attendance was affected by internet connectivity issues for two dyads, but these were resolved after a single consultation with the study team. Asynchronous sessions had greater attendance, suggesting this modality of delivery was more acceptable and accessible for participants. Assessment completion rates were 95% for participants retained in the intervention, suggesting that data collection procedures were feasible and acceptable to participants. However, there were challenges with conducting the SPPB before winter holidays due to scheduling conflicts.

Participants found HACER highly acceptable and satisfactory. Qualitative participant feedback indicated generally positive experiences with HACER. The live, remote delivery for at‐home participation was well received as was the training protocol; participants found it motivating and empowering. Participants reported perceived benefits for both their physical and emotional health, as well as feelings of togetherness or connection with their participating partner and social support from exercise trainers and other individuals with their same lived experience. From qualitative feedback, many participants expressed a perceived improvement in their strength, flexibility, and mobility as well as improved energy levels, emotional well‐being, and self‐confidence. Although the study was not powered to assess efficacy, these perceptions of change in health were supported by our quantitative outcomes for physical activity and physical function.

Our study contributes to the limited research on exercise interventions for Hispanic men with prostate cancer and their caregivers. One other partnered lifestyle intervention has shown feasibility in a sample of men with prostate cancer (50% Hispanic) and their caregiving spouse [57]. In contrast to our intervention, this study was a 12‐week diet and physical activity health coaching intervention delivered once weekly by phone that demonstrated an increase in moderate‐to‐vigorous physical activity, but the effects on resistance exercise and physical function were not explored. Our study emphasized resistance training, an important exercise modality for treatment toxicities common in prostate cancer survivors [20], through partnered exercise, with findings similar to other trials of *Exercising Together^©^*. While a fully powered RCT of *Exercising Together^©^* among prostate and breast cancer survivors and their spouses is pending [24], early phase studies of the intervention have demonstrated feasibility and positive effects on physical, mental, and relationship health. Physical activity level increased among a sample of primarily NHW prostate cancer survivor‐spouse dyads participating in a pilot randomized controlled trial (RCT) of *Exercising Together^©^* [27]. However, effects on physical function and depression were smaller than those observed in our single‐arm HACER pilot trial, which demonstrated an effect on physical function with half the duration of exercise (12 weeks vs. 6 months). A pilot study of *Exercising Together^©^* for NHW prostate cancer survivors and spouses during radiation therapy had similar effects on physical function during 6–8 weeks of exercise [26]. Our cohort of post‐treatment Hispanic men with prostate cancer had lower baseline SPPB scores than the radiation therapy participants (mean: 7.3 vs. 10.5), but a similar observed magnitude of improvement and effect from intervention on physical function.

### 4.1 Study Limitations

This study was limited by the small sample size inherent to feasibility trials. Conducted in Southern Arizona with most participants local to the area combined with our focus on recruiting Hispanic men with prostate cancer, our findings may not be generalizable to other populations or survivorship groups. This trial was a single‐arm design which was sufficient for calculating the effect size of the intervention but was lacking in a control group limiting our ability to determine the efficacy of the intervention on physical and psychosocial health outcomes. We also relied on self‐reported outcomes, which could introduce bias. Participant intention to continue to exercise after the intervention was not asked. However, the online videos continued to be accessed and viewed after the intervention end date suggesting that some participants continued to engage in the exercises at home.

### 4.2 Clinical Implications

Although preliminary, participant responses suggest that HACER had strong signs of improving physical and psychosocial health for dyads. There was evidence of individualized response to increased physical activity with a greater improvement in physical health for survivors nearing clinical significance, while caregivers experienced greater benefits for psychosocial health, with clinically significant improvement in depressive symptoms. The lower level of physical function in our post‐treatment cohort suggests that physical function declined during curative cancer treatment, compared to survivors starting exercise at the beginning of cancer treatment. This is consistent with the broader literature that physical functioning is impaired by prostate cancer treatment and that Hispanic men report worse physical functioning than NHW men after prostate cancer treatment [58, 59]. The contrast in effects on physical and psychosocial health outcomes highlights the potential for HACER to intervene on respective symptoms experienced by survivors and caregivers simultaneously through partnered resistance training, without having to administer different interventions.

### 4.3 Future Directions

We pilot tested a 12‐week culturally adapted intervention with multiple components which included ethnically and gender matched interventionists, bilingual and remote synchronous and asynchronous delivery, and provision of exercise equipment. While not captured in the present study, adding a follow‐up assessment 3–6 months after the active intervention period could determine the sustainability of exercise and effects. Optimization methods may identify which components of the intervention are most effective for targeted outcomes [60]. Further, cost‐effectiveness analysis could inform the large‐scale implementation of the program across various geographic locations and sociodemographic backgrounds [61, 62]. Future research may consider providing flexible scheduling and refining remote access protocols. While all participants utilized their own device with a camera and internet access to attend classes, available technology and internet speed may be barriers to participation; therefore, providing internet enabled tablets or laptops could increase participation and reach. Our study was in a sample of Hispanic men with prostate cancer, but the intervention may have benefits for other groups of cancer survivors and their caregivers, which warrants future investigation.

## 5 Conclusion

HACER was a feasible and acceptable intervention for Hispanic men with prostate cancer and their identified caregivers. HACER has promise for scalability as an efficient live, remote intervention with potential to significantly impact the physical and psychosocial health outcomes of both survivors and caregivers.

## Author Contributions

**Meghan B. Skiba:** conceptualization (lead), formal analysis (lead), funding acquisition (lead), investigation (supporting), methodology (equal), resources (equal), supervision (lead), writing – original draft (lead). **Juan Contreras II:** data curation (supporting), project administration (supporting), writing – review and editing (equal). **Marjorie A. Nelson:** data curation (equal), investigation (equal), project administration (lead), writing – original draft (equal). **Alejandro Recio‐Boiles:** resources (equal), writing – review and editing (equal). **David O. Garcia:** conceptualization (supporting), writing – review and editing (equal). **Floyd H. Chilton:** conceptualization (supporting), writing – review and editing (equal). **Chris Segrin:** formal analysis (supporting), writing – review and editing (equal). **Terry A. Badger:** conceptualization (supporting), resources (equal), supervision (supporting), writing – review and editing (equal). **Kerri M. Winters‐Stone:** conceptualization (lead), methodology (equal), resources (equal), writing – review and editing (equal).

## Funding

This research was funded by an American Cancer Society (Institutional Cancer Research Grant number IRG‐18‐161‐40 to M.B.S.). This research was supported by the Behavioral Measurement and Interventions Shared Resource at the University of Arizona Cancer Center (P30 CA023074). This research was also supported by a National Cancer Institute award to KWS (R01CA218093). Funders had no role in the study design, data collection, analysis, or decision to publish.

## Ethics Statement

This study was performed in line with the principles of the Declaration of Helsinki. Approval was granted by the Institutional Review Board at the University of Arizona (STUDY00003138) on September 25, 2023.

## Consent

Informed consent was obtained from all individual participants included in the study.

## Conflicts of Interest

The authors declare no conflicts of interest.

## Data Availability

The data that support the findings of this study are available on request from the corresponding author. The data are not publicly available due to privacy or ethical restrictions.
